# New Technologies for Influenza Vaccines

**DOI:** 10.3390/microorganisms8111745

**Published:** 2020-11-06

**Authors:** Steven Rockman, Karen L. Laurie, Simone Parkes, Adam Wheatley, Ian G. Barr

**Affiliations:** 1 Technical Development, Seqirus Ltd, Parkville, Victoria 3052, Australia; Steve.Rockman@seqirus.com (S.R.); Simone.Parkes@seqirus.com (S.P.); 2 Department of Immunology and Microbiology, The University of Melbourne, Parkville, Victoria 3052, Australia; a.wheatley@unimelb.edu.au (A.W.); ian.barr@influenzacentre.org (I.G.B.); 3 WHO Collaborating Centre for Reference and Research on Influenza, VIDRL, The Peter Doherty Institute for Infection and Immunity, Parkville, Victoria 3052, Australia

**Keywords:** influenza, vaccine, universal vaccine, cell-culture, egg-based influenza vaccines, cell-based influenza vaccines, recombinant vaccines, mRNA vaccines

## Abstract

Vaccine development has been hampered by the long lead times and the high cost required to reach the market. The 2020 pandemic, caused by a new coronavirus (SARS-CoV-2) that was first reported in late 2019, has seen unprecedented rapid activity to generate a vaccine, which belies the traditional vaccine development cycle. Critically, much of this progress has been leveraged off existing technologies, many of which had their beginnings in influenza vaccine development. This commentary outlines the most promising of the next generation of non-egg-based influenza vaccines including new manufacturing platforms, structure-based antigen design/computational biology, protein-based vaccines including recombinant technologies, nanoparticles, gene- and vector-based technologies, as well as an update on activities around a universal influenza vaccine.

## 1. Introduction

The global burden of influenza is substantial in terms of mortality, morbidity and economic disruption. Seasonal influenza viruses circulate annually worldwide, resulting in 3–5 million cases of severe disease and 300,000–650,000 deaths [1,2]. While all age groups can be affected, the disease is most severe in those at the extremes of life, i.e., young children and the elderly, and those with co-morbidities such as compromised immunity, diabetes, obesity and cardiac disease [3]. Occasionally, non-seasonal infection events can occur, whereby novel influenza A viruses can enter the human population and give rise to influenza pandemics. Over the past 100 years, five such events have occurred in 1918 (H1N1), 1957 (H2N2), 1968 (H3N2), 1977 (H1N1) and 2009 (H1N1), claiming millions of lives [4]. Of further concern are the limited outbreaks of zoonotic influenza strains that have threatened the human population with non-sustained human-to-human transmission arising from virus strains from avian or swine sources, most notably since 1997 [5].

Vaccination is considered the best approach to prevent infection with influenza virus [6]. The very first monovalent virus vaccine (influenza A) was developed in 1938 by the US defence forces, followed by the first bivalent vaccine in 1942 in response to the discovery of influenza B [7]. Trivalent vaccine was introduced in 1978 (typically two influenza A strains and one influenza B strain), followed by a quadrivalent vaccine first registered in the US in 2012 (two influenza A and two influenza B strains) [8]. From 1938 until the 2010s, all registered influenza vaccines were generated in embryonated hens’ eggs and used seed viruses propagated in embryonated hens’ eggs to produce either inactivated vaccine formulations or live attenuated vaccines (LAIVs) [8].

**Figure 1:**
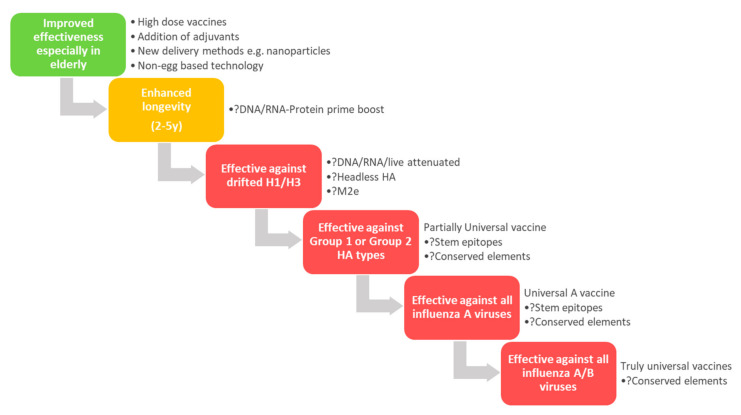
Potential steps and technologies to improve influenza vaccines. Stepwise improvements that might lead to the eventual generation of an effective universal influenza vaccine. Achieved (green), partially achieved (yellow) and in-development/not achieved (red). Listed beside the steps as dot points are strategies that might be used to achieve these outcomes, with question marks indicating strategies that are still in development or have not achieved this outcome to date.

Traditional influenza vaccine approaches have good safety profiles, acceptable tolerability but only moderate efficacy. For example, a meta-analysis of trivalent inactivated influenza vaccines estimated an average efficacy of 59% (95% CI 51–67) [9]. Inactivated vaccines (whole, split or subunit formulations) primarily induce protective immunity through antibodies to the surface viral proteins, haemagglutinin (HA) and neuraminidase (NA). When the virus is propagated in embryonated hens’ eggs, the adaptation of the virus to this substrate can often confer mutations that can reduce or divert vaccine protection against the circulating influenza strain [10,11]. For LAIVs, the strategy is to mimic viral infection without the associated pathogenicity induced by wild-type influenza viruses, while still inducing both humoral and cellular protective responses. However, since LAIVs are grown in embryonated hens’ eggs, LAIVs are still prone to these egg adaptations, which may reduce their effectiveness at least for some components of the influenza vaccine, most notably the A(H3N2). More recently, LAIVs displayed reduced effectiveness in the US in the 2013/2014 and 2015/2016 virus seasons [12,13]. This was suggested to be due to the reduced replication of the A(H1N1)pdm09 virus vaccine component [14], which also appeared to have reduced binding to α2,6-linked sialic acid receptors; the α2,6-linked sialic acid receptors are the primary receptors that human influenza viruses utilize to infect cells in the human upper respiratory tract, and the target of the LAIV, where it replicates following intranasal administration [14]. In addition, the influenza virus can circumvent all current vaccine-induced immunity by accumulating point mutations in the HA and NA proteins, modifying antigenic properties of the virus, which leads to escaping recognition by virus-induced antibodies, a process known as antigenic drift [15]. It should also be noted that immunological imprinting, following the first influenza infection/s in childhood, may also impact vaccine effectiveness through altering the immune response to vaccine exposure later in life [16]. To overcome these shortcomings (unwanted egg adaptations, antigenic drift, and waning or poorly immunogenic antibody responses), new technologies are urgently required. These new vaccine strategies should increase human protection to seasonal and potential pandemic influenza, while importantly still maintaining safety and tolerability. Potential ways to overcome these issues with current and future vaccines are listed in Figure 1 and below.

## 2. Influenza Virus Surveillance for Vaccine Preparation 

The large number of influenza A subtypes (18 HA and 11 NA with various combinations of HA and NA) and the two influenza B lineages, combined with the dynamic nature of influenza through acquisition of mutations, requires constant surveillance and analysis. The World Health Organization (WHO) established a network of influenza surveillance laboratories in 1952, which is now known as the Global Influenza Surveillance and Response System (GISRS) that currently encompasses some 143 National Influenza Centres in 113 countries and 6 WHO Collaborating Centres (CC). This network plays a key role in the surveillance of influenza viruses and the isolation of strains in both eggs and cells, as well as providing gene sequences of these viruses [17]. Antigenic and genetic analyses are also performed by the WHO CCs to determine whether new appearing viruses are antigenically different to the current vaccine strain. This assessment is then declared at the two influenza vaccine strain recommendation meetings held in February and September for the Northern Hemisphere (NH) and Southern Hemisphere (SH) vaccines respectively. Following this declaration of strains, either the sequences are utilised to develop recombinant vaccines or inserted into vectors to allow for reverse engineered rescue of the strain or the wildtype viruses are reassorted with high growth parental viruses. In either case, this process allows for the manufacture of current seasonal and pandemic strains.

## 3. Recently Registered Non-Egg Technologies

Egg propagation of human influenza viruses has been the mainstay of influenza vaccine production to date, utilising inactivated virus (whole virus, split, sub-unit) over several decades [8,18] with LAIV joining this group more recently. In the past ten years, several new platforms that do not use egg-based technologies have been developed and have or are currently undergoing clinical trials to become registered influenza vaccines (see Table 1). The more promising of these approaches will be discussed in further detail below. 

### 3.1. Cell Culture Vaccines 

Given the unpredictable timing of influenza outbreaks and the rapid need for vaccine upon the emergence of a novel strain, the WHO has long advocated for alternate platforms to embryonated hens’ eggs for manufacture of influenza virus vaccines [44]. Reasons for this include the substantial lead time that is required to procure a chicken flock for continuous supply of fertilised eggs and the issue that these chicken flocks are typically replaced following seasonal NH and SH vaccine manufacture, thus there are time periods when egg numbers are limited for vaccine manufacture. Additionally, the risks of avian diseases and potential adventitious agents mean that flocks are generally required to be carefully monitored, and for LAIV-type vaccines, these exclusively require the use of ‘specific-pathogen-free’ eggs, as there is no inactivation process for LAIV like there is with inactivated vaccines, to neutralize any potential adventitious agents. Allergy to egg proteins has also been raised as a concern and driven the non-egg based technologies [44,45], even though in reality, allergy to egg proteins following injection/intranasal administration of influenza vaccine is rarely encountered (reported as only 1.3% of all children and 0.2 % of all adults [46]) and the amount of egg proteins (eg ovalbumin) in vaccines is now very low (≤1 µg/0.5 mL dose for traditional egg based influenza vaccines and 0.24 µg/0.2 mL for LAIV [46]). The outbreaks of avian influenza, particularly of highly pathogenic avian influenza (HPAI) A(H5N1) from 1997–2008, drove a significant push for different platforms to be developed for influenza virus vaccine generation, to complement production in embryonated hens’ eggs [47].

Mammalian cell culture has always been an attractive alternative platform for influenza virus vaccine manufacture, as it can be rapidly initiated with cell stocks exponentially expanded beforehand, then infected with virus [10,45]. Established protocols that are used for seasonal influenza vaccine production can generally be used for pandemic influenza production although with HPAI, the HA must be modified using reverse genetics (RG) techniques before large scale vaccine manufacture to protect both the embryonated hens eggs as well as vaccine manufacturing staff [48]. The capacity of vaccine manufacture is governed by the size and number of the cell fermenters that are available (usually around 2000–6000 litres per fermenter) and the yield of the virus strain in production. Currently, there are two vaccine manufacturers with licensed influenza virus vaccines that use the mammalian cell culture platform. Seqirus Limited (Holly Springs, NC, USA) manufactures Flucelvax® Quadrivalent (Flucelvax® TETRA in various markets), a subunit vaccine which is produced in MDCK 33016PF cells [49]. Flucelvax® was first licensed in 2012 in the US, where it is currently available for those 4 years and older. SK Chemicals (Gyeonggi-do, Korea) manufacturers SKYCellflu Trivalent and Quadrivalent, subunit vaccines produced in MDCK-Sky3851 cells [50] that are licensed in South Korea for 6 months and above and in Malaysia and Thailand for 3 years and above [51,52]. 

Manufacturers of influenza virus vaccines in mammalian cell culture have generally used egg-derived seed viruses but more recently manufacturers have the option of using cell-derived seed viruses which offers a significant advantage to the vaccine recipient. Egg-grown influenza viruses (including egg-derived seed viruses) often acquire mutations in the HA that have a detrimental effect on important antibody epitopes in the HA protein. Strictly cell-grown influenza viruses (those that use cell-seed viruses and are then grown up at scale in cell culture production systems) generally do not acquire these mutations in the HA protein that occur in egg-based systems (or when egg-seed viruses are used in cell culture production systems). Hence these cell-derived vaccines are more antigenically similar to influenza viruses currently circulating in the human population [53], Peck, Barr, Laurie, Rockman manuscript in preparation). Egg adaptation of influenza viruses has been shown to contribute to reduced vaccine effectiveness, as was demonstrated with A(H3N2) viruses in Canada in the 2012/2013 season [54] and the US in the 2017/2018 season [10,11]. Originally, cell-grown vaccines used egg-derived candidate vaccine viruses (CVVs), but since 2017, cell-grown CVVs have also been available for manufacture, enabling the isolation and manufacture of influenza viruses involving only mammalian cells [49]. The benefit of a strictly cell-passaged virus was demonstrated in the 2017/2018 Northern Hemisphere season, which was dominated by A(H3N2) viruses, where Flucelvax® Quadrivalent was found to be significantly more effective than egg-passaged influenza vaccine in preventing influenza like illness (ILI) [10]. Only the A(H3N2) component of Flucelvax® Quadrivalent was isolated and manufactured exclusively in cells [55] for this 2017/2018 vaccine. Since the US 2019-2020 influenza season, all four influenza viruses used in Flucelvax® Quadrivalent have been isolated and propagated exclusively in mammalian cells. Hence it is expected that use of all cell-grown virus vaccines will further improve vaccine effectiveness in future years.

The growth of egg-derived LAIV seeds in mammalian cells may also have the advantage of reducing the need for large numbers of SPF eggs which are difficult to source in many Asian countries, especially in the face of an influenza pandemic [56]. A full risk assessment and safety evaluation to qualify the cell line as suitable for use in man is required, as there is no inactivation step in LAIV production to treat adventitious agents. Unfortunately growing egg-derived LAIV seeds in mammalian cells will not alter the egg adapted changes that are present in the HA, as these largely remain even when egg-derived viruses are passaged in cell culture. Other approaches have also been investigated to improve LAIV performance from codon-optimisation to alternative attenuation methods (e.g. by truncating the *NS1* gene) and other molecular enhancements [57].

### 3.2. Recombinant Protein 

Currently only a single recombinant protein-based influenza vaccine has been registered worldwide although several others have completed or are undergoing phase III large scale human trials. Flublok^TM^, originally produced by Protein Sciences Corporation, Meriden, CT, USA (recently purchased by Sanofi) was licensed by the US Food and Drug Administration (FDA) in January 2013, initially for the prevention of influenza in adults 18–49 years of age. This was expanded in October 2014 to include everyone over 18 years and subsequently a nine-month shelf life was also approved in June 2016. Unlike other influenza vaccines, Flublok^TM^ contains only a single viral protein, HA, but the licensed vaccine contains three times more recombinant HA antigen (45 vs 15 μg) compared to conventional inactivated adult and childhood influenza vaccines. The recombinant HA protein is produced in a proprietary non-transformed, non-tumorigenic continuous cell line (expresSF+ insect cells) grown in serum- free medium, derived from Sf9 cells of the fall armyworm, *Spodoptera frugiperda*. The HAs are expressed in this insect cell line using the baculovirus *Autographa californica nuclear polyhedrosis* virus and the individual HAs are extracted from the cells with buffer and detergent, further purified by column chromatography and formulated into the final vaccine [58]. In a randomized, double-blind, multicentre trial of Flublok^TM^ conducted in the US in 2014-5, a single dose of quadrivalent seasonal influenza vaccine containing 4 recombinant HA’s (RIV4) (45μg of recombinant HA per strain, 180 μg of HA protein per dose) was compared to a single dose of standard-dose quadrivalent inactivated egg-derived influenza vaccine (IIV4) (15 μg of HA per strain, 60 μg of HA protein per dose). The trial compared the relative vaccine efficacy against reverse-transcriptase polymerase-chain-reaction (RT-PCR)–confirmed, protocol-defined, influenza-like illness caused by any influenza strain starting 14 days or more after vaccination, in adults who were 50 years of age or older. A 30% reduction in RT-PCR confirmed influenza was achieved with the RIV4 vaccine compared to the IIV4 vaccine, mainly due to the improved protection afforded by the H3 HA component, as the protection against influenza B was similar with both vaccines [59]. 

Other recombinant HA protein-based influenza vaccines in late stage clinical trials include one produced in plants (Medicago, Quebec City, QC, Canada) and another baculovirus-based nanoparticle vaccine (Novavax, Gaithersburg, MD, USA) that includes a proprietary adjuvant termed Matrix M^TM^. Results of two Phase II clinical trials by Medicago with the plant-based vaccine in 18–49 year olds or over 50 year old subjects, both who received 15, 30 or 60 μg/strain of a HA-bearing quadrivalent virus-like particle (QVLP) vaccine or placebo have been published [60]. They found that the younger aged group responded better to higher doses of HA, with lower responses generated in the older age group, and little benefit with the addition of alum. A 30 μg HA/strain vaccine formulation (MT-2271) was therefore used in two subsequent Phase III trials [60] that have recently been published [61]. The trial in adults (18–64y) found that while MT-2271 was efficacious when compared to placebo for the prevention of infection, it did not meet its primary end point of 70% absolute vaccine efficacy. However when tested in the elderly (>65y), MT-2271 was found to be efficacious and met the pre-specified primary end point of non-inferiority in efficacy compared to a licensed egg-derived vaccine in elderly subjects [61]. Medicago has reported that no application for approval for MT-2271 is currently to be sought for the USA, while an application for review had been accepted by Health Canada. Furthermore, Medicago has stated that they will now investigate the development of their influenza vaccine with the addition of an adjuvant [62].

As detailed above, both of these recombinant protein based vaccines include higher levels of HA per strain in these vaccines (45 µg FluBlok^TM^ and 30 µg MT-2271) compared to split virion and subunit vaccines (15 µg) and this may account for the improved response seen with FluBlok^TM^ [59]. The requirement for the increased HA amounts in these vaccines has not been fully detailed but may be due to different glycosylation motifs generated by insect cells or possibly some levels of aggregation, incorrectly folded or degraded HA that are generated with this platform. The lack of NA in these current baculovirus-based vaccines is also a notable difference from conventional influenza vaccines (inactivated and LAIV) and may also contribute to the need for higher levels of HA as there is no additive protection being generated by the immune response to NA, which has been shown in animal models to enhance protection [63].

## 4. New Technologies

### 4.1. Nucleic Acid Technologies 

Nucleic acid platforms represent an attractive vaccine approach as they have a consistent production and formulation componentry regardless of the vaccine being developed. Manufacturers are able to vary the targeted antigen by simply altering the sequence encoding an antigen or antigens that are to be used in the vaccine. With the development of the ability to synthetically produce GMP grade vaccines using chemical rather than biological processes, nucleic acid-based vaccines have become attractive due to the ease of production, high quality, short lead-time for availability, adaptability to encode different antigens, reduced risk of adventitious agents and cost-effective production. However, each platform, DNA, RNA, modified RNA and self-amplifying RNA, has its own inherent challenges.

#### 4.1.1. DNA

DNA vaccination has been developed and researched for over the past three decades [64,65]. DNA based vaccines use recombinant technology to clone complementary DNA sequences that encode the antigen sequence of interest into eukaryotic expression plasmids. These vaccine plasmids are amplified in bacteria, purified and then inoculated into recipients by various methods including injection, gene-gun and electroporation [66,67]. DNA vaccines in this format are non-infectious, non-replicating and do not induce vector-specific immunity [68]. The immunological advantage of DNA vaccines is the ability to present the immunogen to both major histocompatibility complexes (MHC) class I and class II molecules inducing both humoral and CD4+ and CD8+ T cell responses. The ease of construction of DNA vaccines and induction of different arms of the immune response differentiates DNA vaccines from currently licensed inactivated vaccines which have longer lead times and generate only humoral and CD4+ T cell responses. Thus DNA vaccines offer an attractive approach to combat influenza (reviewed in [69]) which was one of the first diseases to be targeted with this technology.

Studies assessing use of DNA vaccines to prevent influenza have been conducted using direct injection of DNA. Mice injected with DNA encoding nucleoprotein (NP) and HA of influenza were protected from heterologous and homologous challenge respectively [70]. Heterologous protection was further demonstrated in chickens, ferrets and non-human primates [71,72]. Naked DNA vaccine approaches led to both humoral and T cell mediated responses; antibody alone was unable to protect mice from lethal challenge [73] indicating that cell-mediated immunity was required for protection. However, while producing potent immune response in animal models, especially in mice, the induction of immunity in humans with DNA vaccines has been relatively poor [74]. This may be due to the degradation of DNA by host enzymes meaning even larger doses of DNA may be needed in man, or the ability for DNA to enter the nucleus is lacking leading to poor synthesis of the viral protein(s) and reduced immune responses [74]. Further there is some concern around DNA vaccines with respect to the risk of integration of DNA into the recipient’s genome though this unlikely [75]. Priming with DNA and then boosting with a conventional monovalent H5 vaccine was shown to improve immune responses [76] but was not effective in adults using seasonal influenza strains, possibly due to pre-existing immunity to the priming, unmatched prime and boost antigens, or the lengthy (36 week) interval between the prime and the booster doses [77]. A phase I clinical study in children and adolescents (6–17 years old) priming with trivalent DNA vaccine and boosting with trivalent inactivated vaccine showed the strategy was safe and well tolerated, though the needle-free jet injection device (Biojector 2000) was associated with increased pain, swelling and redness as compared with conventional need and syringe administration. Overall, antibody responses were consistent, yet there was a significant increase in antibodies to the H1N1 component of the vaccine in participants that received the DNA-IIV3 vaccine as compared to those that received two doses of IIV3 [78]. Currently there is little activity in the development of influenza DNA vaccines and to date no DNA vaccine has been approved for use in humans.

#### 4.1.2. RNA

To date, a number of approaches to utilise RNA as an influenza vaccine have been developed. Initial studies of naked unmodified RNA delivered via the intramuscular route has demonstrated only modest protection against influenza [79]. From these studies re-formulation of mRNA to improve stability, such as incorporating the RNA into nanoparticles or liposomes, may also improve delivery of the mRNA intracellularly so the viral protein can be produced, processed and an antigen-specific immune response generated more readily. The current RNA vaccine approaches include non-replicating mRNA and in vivo self-replicating or amplifying mRNA. In vitro dendritic cell non-replicating mRNA vaccines are an alternative approach yet are best suited to specialist applications such as cancer therapy due to extensive in vitro manipulation, the high cost and the difficulty scaling up this approach for an influenza vaccine.

Non-replicating mRNA: Non-replicating mRNA is the simplest type of mRNA where the mRNA is taken up by the recipients’ cells which then translate the antigen of interest. The advantages of non-replicating mRNA vaccines, that is the ease of construction and the small size (2–3 kb), are offset by a shorter half-life and low level protein expression [80]. The seminal study of vaccination with unmodified mRNA for influenza demonstrated induction of cytotoxic T cells [81]. Protection was then demonstrated after intradermal inoculation in mice, ferrets and pigs [82] and T cell induction after intravenous immunization of mice [83]. Encapsidating the RNA in a lipid nanoparticle carrier (LNP) enabled greater efficiency of cellular uptake and in vivo protein translation [84]. LNP formulated with unmodified mRNA immunised intramuscularly into non-human primates subsequently demonstrated equivalent antibody responses after the first dose, and superior antibody responses following a second dose compared to an adjuvanted subunit influenza vaccine [85]. Incorporation of modified nucleosides such as pseudouridines into the mRNA has led to claims of higher translational capacity or stability of mRNA vaccines [86]. Nucleoside modified mRNA-LNP vaccines have demonstrated encouraging immune responses in mice, ferrets and non-human primates via intramuscular and intradermal delivery [87,88,89,90]. This is the approach of Moderna Therapeutics (see Table 1) who have published Phase I clinical trial data with an avian A(H10N8)-formulated modified mRNA lipid nanoparticle vaccine [91]. This clinical trial (NCT03076385) is complete while a second trial (NCT03345043) of A(H7N9) while active, is not recruiting at this time.

Self-amplifying mRNA: Self-amplifying mRNA (sa-mRNA) vaccines incorporate the target antigens with additional non-structural protein-encoding sequences derived from a modified alphavirus genome as a vector [92]. Delivery of such a vector into the cytoplasm allows the translation of RNA-dependent RNA polymerase (RDRP) that results in the transcription of a negative sense copy of the construct. Following the translation of the RDRP, the negative sense RNA serves as a template for positive strand transcription of genomic and subgenomic mRNA. The smaller subgenomic mRNAs are then transcribed at higher levels resulting in increased levels of antigen transcription/expression and thus the self-amplification of the vaccine antigen. The ability to self-replicate the antigen coding RNA intracellularly, with a resultant increase in antigen expression, enables a smaller dose of RNA to be used than with mRNA alone. For example, sa-mRNA vaccines can reduce the level of RNA by formulation with LNPs from 10 µg (partial protection with homologous lethal challenge in mice) [79] to smaller doses of 0.1 to 1 µg to achieve complete protection with a HA-sa-mRNA encoded LNP [93]. Direct comparison of sa-mRNA versus a non-replicating mRNA in mice indicated a 64-fold lower level of RNA with similar levels of protection [94]. Non-HA administration of sa-mRNA-LNP vaccines that include NP, M1 and NP+M1 resulted in T cell responses, yet these were only partially protective in mice [95]. Further development of modified formulations to explore more efficient delivery vehicles include Chitosan containing LNP or polethyleneamine [96,97] and chemically modified ionizable dendrimer LNPs [98]. At this time, sa-mRNA-based vaccines for influenza have not reached the clinic but have been applied to the recent coronavirus vaccine pandemic [99,100]. The challenge with mRNA vaccines is the unintended effects of the introduction of mRNA into cells with detection by toll like-receptors (TLRs) in the host. Thus, delivery of the mRNA must be efficient since RNA can easily be broken down both intra- and extracellularly. In addition, mRNA vaccine, like most conventional vaccines, requires refrigeration or freezing for stability, especially as RNA is inherently more easily degraded than protein-based vaccines. While there are a number of companies that have generated preclinical and proof-of-concept data with the various RNA strategies, many of these programs have been put on hold due to the COVID-19 pandemic with the focus on generating vaccines directed to this new virus. A notable outcome from the activities around COVID-19 directed RNA vaccines is the opportunity for the rapid clinical pathway for sa-mRNA vaccines due to regulators’ consideration that the safety profile is similar to RNA that may be directed to a different infectious agent (such as influenza) through the simple change of the viral gene sequence.

### 4.2. Viral Vectors 

#### 4.2.1. Modified Vaccinia Ankara (MVA) and Pox Viruses

Recombinant pox viral vectors have been (i) used for boosting the immune response alongside numerous other vectors licensed for use against a variety of animal viruses and (ii) investigated for carrying transgenes for various human vaccines [101]. Vaccitech (a spin-out company of the University of Oxford) has developed an MVA vaccine that was hoped to be a potential pandemic universal influenza A vaccine candidate (MVA-NP+M1 (VTP-100)). It contained conserved influenza antigens nucleoprotein (NP) and matrix 1 (M1) as a fusion protein and was designed to protect against all strains of influenza A virus, including swine, avian and human influenza [102]. A phase 2b study using this vaccine with 118 participants in a randomized, double-blind, placebo-controlled, influenza challenge study (using the challenge virus A/Belgium/4217/2015 (H3N2)) failed to reach its primary endpoint of a thirty percent reduction in overall viral shedding. A larger phase 2b trial, FLU009 with 2149 subjects, tested VTP-100 given as an adjunct to current licensed quadrivalent influenza seasonal vaccine in an attempt to decrease influenza-like illness [103]. While the vaccine was reported to be safe and well tolerated, the trial was stopped after the first year as it was determined that it was unlikely to meet the primary endpoint and achieve the required level of protection relative to participants receiving the standard quadrivalent influenza vaccine alone [30]. An MVA-based influenza vaccine no longer appears on the Vaccitech website pipeline of products under development [104].

#### 4.2.2. Adenovirus Based Vaccines

Recombinant adenovirus (AdV) as a vector for vaccine development have been studied extensively both pre-clinically and clinically, are well tolerated in vivo, possess a good safety profile and appear to be capable of stimulating potent immune responses to the encoded vaccine antigen. Human adenoviruses such as type 5 serotype (Ad5) have been commonly used in gene therapy applications, but have also been shown to be highly immunogenic, precluding them as a multi-delivery option. This finding and the high seroprevalence of neutralizing antibodies in humans to Ad5 limits its use in vaccines. This has led to the use of rare human AdV’s such as species B serotypes Ad35, or species D AdV’s: Ad26, Ad28 or Ad48, which have low seroprevalence in humans [16,45]. Alternatively, genetically modified chimeric Ad5-based vectors (e.g., Ad5HVR48) which substitute the major antigenic epitopes within the hexon for those derived from rare AdV’s are also under investigation [105]. One of the most advanced adenovirus-based influenza vaccines has been developed by a commercial company Vaxart (San Francisco, CA, USA) which conducted a Phase II trial in 2016-7 (ClinicalTrials.gov, NCT02918006, [106]). Their vaccine, known as VXA-A1.1, is a replication-defective adenovirus type-5 vectored vaccine that expresses the influenza HA from the A(H1N1)pdm09 virus A/California/04/2009 in conjunction with a TLR-3 agonist as an adjuvant [107]. The vaccine was administered orally in specifically formulated oral tablets designed to release the virus in the ileum. Their study was conducted in individuals 18–49 years old that had been prescreened for A/California/H1N1 haemagglutination inhibition (HAI) titres within 120 days of enrolment and were eligible to participate if they had an HAI titre of less than 20 and were in good health. The 179 enrolled individuals were assigned (2:2:1) to receive a single immunisation of either 10^11^ infectious units of VXA-A1.1 (in 7 monovalent tablets vaccine/person) orally plus a placebo IM injection, or a full human dose of quadrivalent inactivated influenza vaccine (IIV; Fluzone containing 15ug HA from A/California/07/2009 (H1N1)pdm09 and the 3 other vaccine components), via an intramuscular injection plus oral placebo tablets, or a full placebo dose (placebo IM injection plus oral placebo tablets). Eligible subjects were then challenged with an A/California/H1N1 2009-like, A(H1N1)pdm09, wild-type influenza A virus, administered intranasally at a dose of 0.5mL (0.25ml per nostril) (9.0 × 10^5^ TCID50/dose) using a mucosal atomizer. Results showed that the orally administered VXA-A1.1 was well tolerated and generated protective immunity against virus shedding, similar to a licensed intramuscular IIV, with influenza-positive illness after challenge detected in 17 (29%) of 58 individuals in the VXA-A1.1 group, 19 (35%) of 54 in the IIV group, and 15 (48%) of 31 in the placebo group [106]. Adenovirus-based vaccine strategies for COVID-19 are also underway at this time. However, it should be noted that the utility of reusing the same AdV serotype for revaccination is still problematic, due to the strong host responses to the vector that are usually observed and in addition concerns have been raised that the use of Ad5 as a vaccine vector may increase the risk of HIV-1 acquisition in certain sub-groups of gay men [108].

### 4.3. Structure/Computational Design, COBRA

Alternative influenza vaccine approaches based on immunity to the head region of the influenza HA that are designed to overcome this variable portion of the molecule include a technology known as computationally optimized broadly reactive antigens or COBRAs. In order to overcome the high variability of the head of influenza HA, this approach generates computationally optimized broadly reactive antigens (COBRAs) for the influenza HA [109]. While vaccines with this technology have shown promise in animal models, they have not progressed to human trials to date. In addition to achieve protection against different subtypes may require a combination of antigens requiring a ‘cocktail’ of COBRA HA’s representing different subtypes, hence complicating any vaccine design using this approach [110].

## 5. Universal Influenza Vaccines

The ongoing quest for an influenza vaccine for broad and lasting immunological protection has seen tremendous technological advancement in recent years usually under the broad umbrella term of “Universal Influenza Vaccines”. Much of the recent focus has been on eliciting antibodies to the stem or stalk domain of HA, a site with broad sequence conservation between influenza subtypes and a proven target of cross-reactive antibody recognition [111]. Recently, clinical assessment of chimeric HA immunogens, comprising an immunologically novel head domain (H5, H8 or similar) and a conserved stalk (H1) was reported [112]. Delivered as either LAIV or as inactivated vaccines, chimeric HA vaccines were able to drive expansion of stalk-specific immunity in volunteers, in addition to concomitant responses against the novel head component. These observations align with previous reports of expanded stem immunity following infection with previously un-encountered strains (such as A(H5N1) or A(H1N1)pdm09) [113] or after administration of novel vaccines (A(H5N1), A(H7N9)) [114,115]. Notably, expanded stalk responses are notoriously transient, suggesting that while pre-existing anti-stalk immunity is widespread in adults in the form of immunological memory [116], it is refractory to further boosting by vaccination. Memory responses are perhaps destined to fail, as recent studies in mice [117] and in influenza vaccinated humans [118] suggest that re-stimulating HA-specific memory B cells is only a pathway to transient antibody production and cannot reliably induce long-term antibody-secreting plasma cells in the bone marrow, critical for life-long vaccine protection. So, where to from here for stalk-based vaccines? It could be that such vaccines will need to be administered to neonates before the onset of childhood influenza infections. However, the pathway to clinical trial and licensure for such a strategy is difficult to imagine. Alternatively, technological improvement to vaccine platforms or immunogens are required to boost the potency and epitope selectivity of elicited humoral responses.

### 5.1. New Epitopes for Rational Immunogen Design

The molecular definition of highly cross-reactive B cell epitopes can inform the design of vaccines for broad protection. Multiple, partially overlapping epitopes in the HA stalk can mediate neutralization and/or in vivo protection spanning pan-group 1 [119], pan-group 2 [120,121], both group 1 and group 2 influenza A [122,123], even extending across influenza A and influenza B viruses [124] (Figure 1). Other major cross-protective sites, that generally mediate only pan-subtype level protection, have been identified. For example, a class of structurally convergent antibodies can mimic the sialic acid receptors of influenza and potently block receptor engagement for a wide range of viral isolates [125,126]. Similarly, broad anti-H1N1 protection can be mediated by antibodies binding an epitope distal from the RBS and classical antigenic sites in the head domain of H1N1, that some have dubbed the “lateral patch”. Exemplified by the mouse antibody 446D1 [127], antibodies binding this site show broad antiviral activity in vitro and in vivo without mediating HAI, and can be readily identified in the human memory B cell compartment [127,128]. Another example is the residual esterase domain of A(H5N1) isolates, which is readily targeted by broadly neutralizing human antibodies for pan-subtype protection [129]. Further, recent studies on influenza B have shown widespread and underappreciated cross-reactivity in the antibody and B cell response to seasonal vaccines between the two antigenic influenza B lineages (B/Yamagata/16/88-lineage and B/Victoria/2/87-lineage) [130,131].

Most recently, a number of exciting new epitopes have been described, some with unique mechanisms of anti-viral activity. A novel epitope at the interface of monomers that make up the trimeric HA spike is conserved across a wide range of influenza A subtypes, and antibody recognition of this epitope can facilitate disruption of the trimer and provide passive protection to challenge animals [132]. Similarly, another interface epitope occluded in the native HA trimer but recognised by B cells in multiple donors was recently reported [133]. Although reconstituted antibodies were non-neutralising in vitro, broad Fc-mediated protection was seen after passive transfer against experimental challenge with diverse viruses. Outside of HA, highly cross-reactive epitopes recently identified in NA can similarly confer broad cross-subtype protection. The recovery of three highly potent, heterosubtypic antibody lineages from humans highlighted the existence of anti-NA antibodies with exceptionally broad protective potential against both influenza A and B viruses [134]. While the growing list of cross-reactive targets in HA and NA is promising, challenges remain in the optimized design of HA and NA immunogens to focus immunity onto these largely subdominant epitopes. In addition, the capacity, frequency and fitness impact of viral escape from antibody neutralization at these sites remains to be assessed.

### 5.2. Modulating Immunogen Glycosylation and Glycan Engineering

A critical role for glycan in modulating vaccine immunogenicity and epitope focus is growing. Glycan interactions with the host mannose-binding lectin (MBL) was recently established to be critical for supporting the robust immunogenicity of multimeric HA vaccines by facilitating retention of vaccine antigens in draining lymph nodes [135]. Interestingly, monoglycosylated forms of HA produced by EndoH digestion induce significantly more cross-protective antibodies in mice including against the HA stem [136], highlighting that glycan shielding plays a key role in occluding recognition of important cross-reactive epitopes. While the removal of glycans within the HA stem domain is known to enhance the elicitation of cross-reactive neutralizing responses [137], it was recently shown that selective and precise glycan positioning can drive dominant pan-group 1 humoral responses against a HA stem-based immunogen toward greater recognition of group1/2 cross-reactive sites, thereby broadening protective immunity [137]. Therefore, many exciting opportunities exist going forward for rational immunogen tailoring through strategic glycan engineering of HA and NA proteins to maximise the immunological visibility of highly cross-protective epitopes. 

### 5.3. New Vaccine Platforms for Broader (Universal) Protection

Finally, next generation vaccine platforms offer unique opportunities to tune vaccine immunogenicity, broaden humoral responses and increase the quality of vaccine-elicited antibody, T and B cell memory responses. While nanoparticle-based vaccine platforms are now a well-established mechanism to boost immunogenicity [138], recent studies have highlighted the potential for rationally designed vaccine approaches. Development of multi-component, self-assembling nanoparticle platforms provide a basis for co-loading different vaccine immunogens onto a single vaccine unit [139], facilitating multi-valent immunity and increasing the likelihood of recognition by cross-reactive B cells in vivo. Building on this principle, Kanekiyo et al assembled a novel mosaic array of HA receptor binding domains from diverse A(H1N1) strains upon a ferritin-based nanoparticle core, and were able to demonstrate the selective promotion of cross-reactive B cell responses in immunized mice resulting in qualitative and quantitative improvements in vaccine elicited antibody [140]. These illustrative examples highlight a way forward for next-generation vaccine design. In particular, the exciting recent demonstration of fully customizable, synthetic protein scaffolds [141] for optimal positioning of vaccine antigens should provide a highly tractable path for fine control of epitope presentation to the immune system, likely critical to the success of a universal influenza vaccine.

### 5.4. Other Viral Proteins as Targets for Universal Vaccines

M2 is a type III membrane protein present on the influenza virion surface, and vaccines based on the highly conserved ectodomain (M2e) have progressed through to phase 2 clinical trials [142]. While antibodies specific for M2e can mediate non-neutralising protection via Fc-dependent antiviral functions, it remains unclear if current vaccine technologies can generate and maintain sufficient titres to be protective in human populations. Another approach is the targeting of NP and other internal viral proteins to promote T-cell immunity. Evidence for a protective role for anti-viral CD4+ or CD8+ T cell responses is widespread in animal models, with indirect confirmation that CD8+ T cells recognising conserved epitopes might confer some protection in humans [143]. While current inactivated vaccines do not elicit significant cellular responses [143], it is possible viral vectored vaccines in pre-clinical development may be sufficiently immunogenic to prime long-lasting protective T cell responses which, while not producing sterilising immunity, may protect from disease and/or onward transmission. A shared challenge for HA-stem, M2e and T cell-based vaccine candidates is that classical HAI assays cannot be used as a benchmark for licensure. Therefore, costly large-scale clinical testing will be required to demonstrate both efficacy and superiority/inferiority to conventional influenza vaccines on the market.

## 6. Conclusions

Influenza continues to inflict a significant burden of disease on both humans and animals despite the ongoing development of multiple vaccine approaches applied to humans over the past eight decades, since the identification of the causative virus. Licensed influenza vaccine approaches have included whole inactivated, detergent or solvent split vaccines, subunit vaccines, recombinant proteins, live attenuated and adjuvanted vaccines delivered by different modalities including intramuscular, intradermal and intranasal routes. None of these approaches has elevated seasonal influenza vaccines to the desired efficacy (>90%) and all still require regular updates to match virus evolution to remain effective. Similar problems exist in the production of broadly cross reactive or universal vaccines that would protect humans against animal or potential pandemic influenza viruses. In order to adequately improve influenza vaccines will require further investigation and extensive human clinical trials of the most promising novel vaccine approaches outlined in this review and, in parallel, a better understanding of the immune response to influenza infections and vaccines and ways to overcome immunosenescence. Further insights into these different platforms may be gained by the storm of activity and large number of human clinical trials that have arisen as a response to the 2020 COVID-19 pandemic, and these endeavours might ultimately also lead to significantly improved influenza vaccines. 

## Figures and Tables

**Table 1:** Non-egg-based Influenza Vaccines in commercial development that have completed human clinical trial(s).

Company	Phase	Administration	Reference
Recombinant			
BiondVax	Phase III	Oral	[19]
Imutex	Phase II	SC	[20]
Recombinant—VLP			
Novavax	Phase III	IM	[21]
Osivax	Phase II	IM	[22]
Medicago	Phase III/discontinued	IM	[23]
Medigen	Phase II	IM	[24]
Recombinant—H5 protein fragment			
Generex	Phase I	Oral	[25]
Live attenuated			
Codagenix	Phase I	Nasal	[26]
FluGen	Phase II	Nasal	[27]
Vivaldi	Phase II	Nasal	[28]
Polymun	Phase I	Nasal	[29]
Vector—adenovirus			
Vaccitech	Phase II	IM	[30]
Vaxart	Phase II	Oral	[31]
Altimmune	Phase II	Nasal	[32]
Vector—alphavirus			
AlphaVax	Phase II	IM	[33]
Adjuvant—novel			
BlueWillow	Phase I	Nasal	[34]
Nitto Denko	Phase I	Sublingual	[35]
Mercia	Phase II	IM	[36]
Adjuvant—toxin			
Mucosis	Phase I	Nasal	[37]
Eurocine	Phase I/II	Nasal	[38]
Advagene	Phase II	Nasal	[39]
mRNA			
Moderna Therapeutics	Phase I	IM	[40]
DNA vaccine			
Inovio	Phase I	IM	[41]
Virosomes			
Mymetics	Phase II	Nasal	[42]
Dendritic cells			
CEL-SCI	Phase I	IM	[43]
